# Negative life events and problematic smartphone use among university students: the mediating role of self-efficacy and the moderating role of physical activity

**DOI:** 10.3389/fpubh.2025.1704120

**Published:** 2026-01-14

**Authors:** Chao Yang, Wenying Huang, Chang Hu, Wen Zhang, Bin Chen, Chi Zhou, Dong Zhu, Bo Xu

**Affiliations:** 1 Physical Education College, Jiangxi Normal University, Nanchang, China; 2 Hunan University of Medicine, Huaihua, Hunan, China

**Keywords:** moderated mediation model, negative life events, physical activity, problematic smartphone use, self-efficacy

## Abstract

**Objectives:**

This study explores how negative life events relate to problematic smartphone use among university students and examines whether self-efficacy acts as a mediator and physical activity as a moderator in this relationship.

**Methods:**

A cross-sectional survey was conducted among undergraduates at eight universities in Nanchang, China, from November 2024 to March 2025. Validated instruments were used to assess negative life events (ASLEC), self-efficacy (GSES), physical activity (PARS), and problematic smartphone use (SAS-SV). The sample included 2,680 participants (47.6% male, 52.4% female; mean age = 19.60 years). Statistical analyses included Pearson correlation analysis and PROCESS models for mediation and moderation.

**Results:**

Negative life events were positively correlated with problematic smartphone use and negatively correlated with self-efficacy and physical activity. Self-efficacy partially mediated the relationship between negative life events and problematic smartphone use [indirect effect *β* = 0.05, 95% CI (0.04, 0.07)], with a significant direct effect. Physical activity attenuated the positive correlation between negative life events and problematic smartphone use (interaction *β* = −0.01, *p* < 0.001).

**Conclusions:**

Negative life events, self-efficacy, physical activity, and problematic smartphone use are interrelated among university students. Enhancing self-efficacy and promoting physical activity may help mitigate problematic smartphone use. However, the cross-sectional design and reliance on self-reports limit causal inference.

## 1 Introduction

Problematic smartphone use has increasingly become a significant behavioral health concern among university students and is closely linked to poorer academic performance, sleep disturbances, impaired social functioning, and heightened psychological distress (1–3). Approximately 25% of university students screen positive for problematic smartphone use. This population faces unique developmental challenges, including sustained academic pressure, shifting peer relationships, and identity formation, which heighten stress exposure and the likelihood of maladaptive coping (4–6). Negative life events such as examination failure, interpersonal conflict, and financial strain are also common during this period (7–9) and have been consistently associated with elevated anxiety, depressive symptoms, emotion dysregulation, and problematic smartphone use (10, 11).

Although prior studies have established the association between negative life events and problematic smartphone use, the underlying mechanisms remain insufficiently clarified (12). Much of the existing literature has focused on direct effects, with fewer studies examining the joint roles of internal psychological resources and adaptive behaviors in this association. In addition, prior research has rarely tested moderated mediation models integrating both self-efficacy and physical activity, and findings among university students, a population at particularly high risk, remain inconsistent. These gaps highlight the need for a more comprehensive model to explain how and when negative life events contribute to problematic smartphone use.

Several theoretical perspectives provide a foundation for understanding these processes. Social Cognitive Theory emphasizes self-efficacy as a central determinant of coping choices and behavioral regulation (13, 14). Conservation of Resources Theory further suggests that stress arising from negative life events depletes psychological resources, such as self-efficacy, thereby increasing vulnerability to maladaptive behaviors (15). Behavioral Activation Theory complements these perspectives by proposing that engagement in meaningful and rewarding activities, such as physical activity, can counteract negative affect and disrupt maladaptive patterns (16, 17). Together, these theories suggest an integrated pathway in which negative life events reduce self-efficacy, thereby increasing problematic smartphone use, while physical activity may buffer these effects. Building on these theoretical and empirical gaps, the present study examines whether self-efficacy mediates the relationship between negative life events and problematic smartphone use and whether physical activity moderates this pathway. This approach provides a multidimensional understanding of how psychological and behavioral factors jointly shape digital health risks among university students.

### 1.1 Negative life events and problematic smartphone use among university students

Negative life events refer to experiences that generate psychological stress and adverse emotions, such as examination failure and interpersonal conflict (18). Among university students, these events are closely associated with anxiety and depression, and they substantially increase the risk of problematic smartphone use (19). Prior research consistently shows a significant positive association between negative life events and problematic smartphone use (20). The primary pathway can be understood through emotion regulation and coping selection. Negative life events elicit distress, and smartphones, as readily accessible tools that provide immediate feedback through entertainment and social features, offer short-term relief and distraction, which strengthens reliance on the device (21). Negative life events can also disrupt daily routines and academic structure, making students more likely to adopt problematic smartphone use as a short-term coping strategy that is costly in the long term (22). In contemporary campus settings, the high accessibility and constant availability of smartphones facilitate rapid psychological comfort through social media, gaming, and streaming, heightening the risk of excessive use, neglect of academic and sleep responsibilities, and reduced face-to-face interaction (23). In addition, frequent notifications and social comparison repeatedly activate cues related to stressors, maintain negative affect and cognitive load, and further magnify the impact of negative life events on problematic smartphone use (24, 25).

### 1.2 The mediating role of self-efficacy

External stress often influences behavioral outcomes by altering individuals' internal psychological resources (26). Self-efficacy refers to one's belief in the ability to organize and execute actions to achieve goals, reflecting expectations of control over difficult tasks and willingness to persist (27). Conservation of resources theory and social cognitive theory both emphasize that self-efficacy is a key internal resource that can buffer stress and sustain goal-directed behavior (28). When negative life events accumulate, experiences of failure and uncertainty undermine individuals' sense of competence in academics and self-regulation, leading to the depletion and decline of self-efficacy (29). Once self-efficacy is reduced, individuals find it more difficult to mobilize executive functions and self-control to face tasks, and they tend to prefer immediate, low-cost compensatory regulation strategies, such as using smartphones for brief comfort and distraction, which increases the risk of problematic smartphone use (30, 31).

At the same time, low self-efficacy is regarded as a vulnerability factor for various maladaptive behaviors (32). Research shows that diminished self-efficacy increases the perceived difficulty of tasks and lowers expectations for persistence and delay of gratification, thereby increasing the likelihood of choosing short-term reinforcing behaviors, such as passive scrolling, social media immersion, and gaming, as a form of avoidance (33, 34). In contrast, higher self-efficacy facilitates constructive coping, including time management, help-seeking, and strategic learning, thereby reducing the need to use smartphones for escape at its source (35). Therefore, self-efficacy is likely to serve as a critical mediating mechanism linking negative life events to problematic smartphone use (36).

### 1.3 The moderating role of physical activity

The relationship between negative life events and problematic smartphone use is complex and multidimensional, and it may be moderated by key individual and contextual resources (37). Physical activity refers to planned or routine bodily movements undertaken to improve or maintain physical and mental health, and it confers multiple benefits, including mood enhancement, improved cognitive control, and better sleep (38). Prior research has identified physical activity as an important factor that moderates psychological adaptation, protecting against stress and negative affect (39, 40). Regular physical activity can function as a protective factor by reducing the intensity and duration of anxiety and depressive symptoms, thereby lowering the likelihood of maladaptive coping (41, 42).

When individuals maintain higher levels of physical activity, they are more likely to cope with stress in constructive ways, exhibit better attentional control and emotion regulation, and show less reliance on immediate rewards, which in turn reduces the tendency to use smartphones primarily for escape and mood repair (43, 44). In contrast, when physical activity levels are low, negative life events are more likely to elicit persistent negative emotions and cognitive load, and individuals are more prone to seek short-term comfort through passive screen use, which elevates the risk of problematic smartphone use (45, 46). Physical activity can therefore be viewed as a protective resource that mitigates the adverse consequences of negative life events (47).

According to behavioral activation theory, physical activity helps individuals restore psychological and physiological balance in the face of adversity and reduces the likelihood of problem behaviors (48). Negative life events typically increase perceived stress and negative affect, thereby increasing the likelihood of engaging in problematic smartphone behaviors to avoid and regulate emotions (25). Physical activity moderates this process through two primary pathways. First, at the physiological level, it promotes the release of dopamine and endorphins, improves the emotional baseline, and lowers the need for immediate digital stimulation (49, 50). Second, at the psychological and behavioral levels, it enhances self-efficacy, provides structured time and offline social opportunities, and reduces windows and triggers for passive scrolling, thereby weakening the link between stress and problematic smartphone use (51, 52).

### 1.4 Current study

In summary, this study proposes the following hypotheses for university students:

**Hypothesis 1**. There is a significant association between negative life events and problematic smartphone use.

**Hypothesis 2**. Self-efficacy mediates the relationship between negative life events and problematic smartphone use.

**Hypothesis 3**. Physical activity moderates both the direct link between negative life events and problematic smartphone use and the second stage of the mediating pathway via self-efficacy, forming a moderated mediation model.

**Figure 1:**
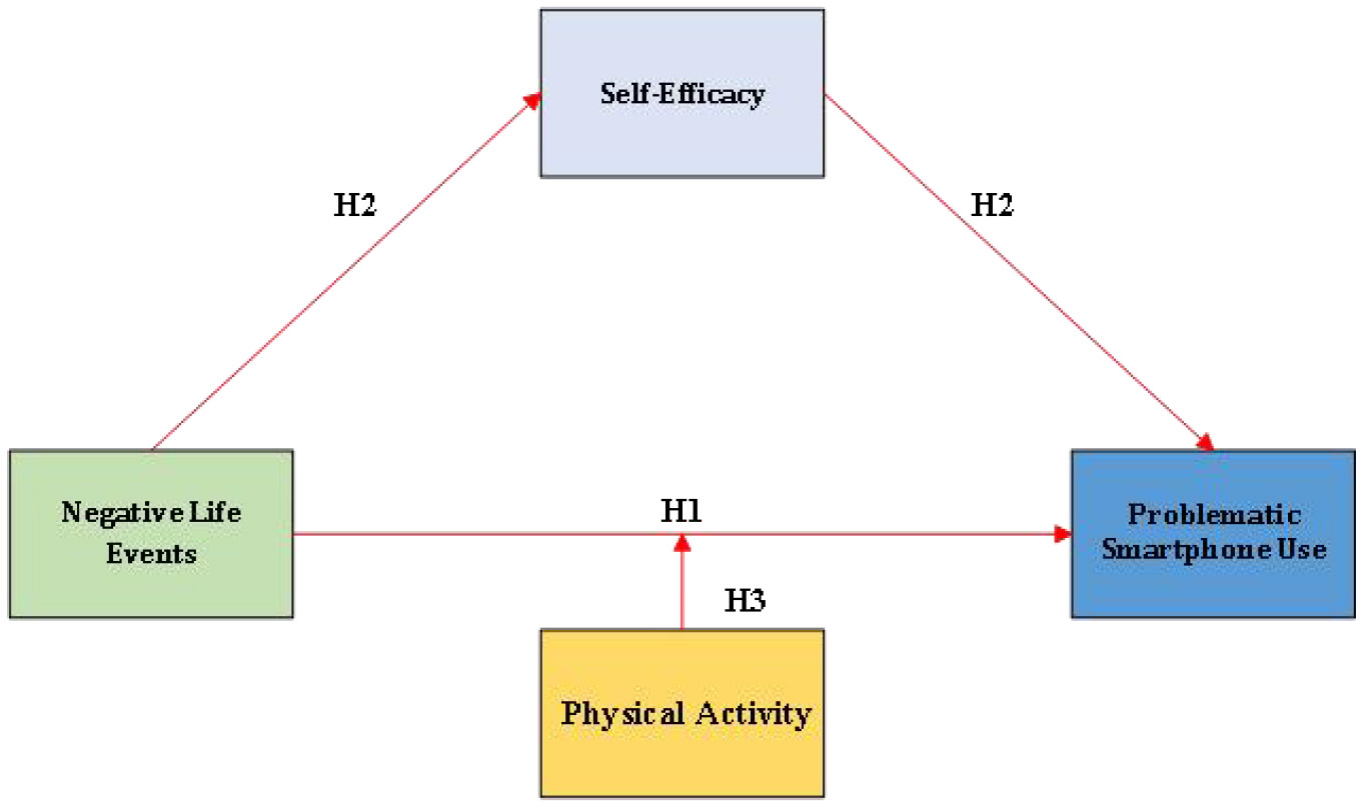
Moderated mediation model.

The findings are expected to clarify how these factors interact among university students. Such insights can inform the design of interventions that reduce problematic smartphone use by enhancing self-efficacy and promoting physical activity. The hypothesized model is shown in Figure 1.

## 2 Participants and methods

### 2.1 Participants

This cross-sectional survey was conducted from November 2024 to March 2025 in Nanchang, Jiangxi Province. Eight universities participated in the study. Although stratified cluster random sampling was applied within each institution, the selection of the eight universities themselves was based on convenience, determined by feasibility, institutional collaboration, and willingness to participate rather than random selection. This approach should be considered when interpreting the representativeness of the sample. Using a stratified cluster random sampling strategy, undergraduate classes were randomly selected within each university, stratified by discipline category (medical, pedagogical, and comprehensive). The sampling frame was built from departmental rosters, and classes were selected using computer-generated random numbers; to reduce homogeneous clustering bias, no more than two adjacent grades were sampled consecutively within the same department.

The survey was administered on the Wenjuanxing platform (https://www.wjx.cn/), with links/QR codes distributed by instructors or counselors during post-class sessions or class meetings. Before starting, participants read and signed an online electronic informed consent form, which clarified the study purpose, voluntariness, the right to withdraw at any time, anonymity, and data use; they then completed a self-administered questionnaire.

**Figure 2:**
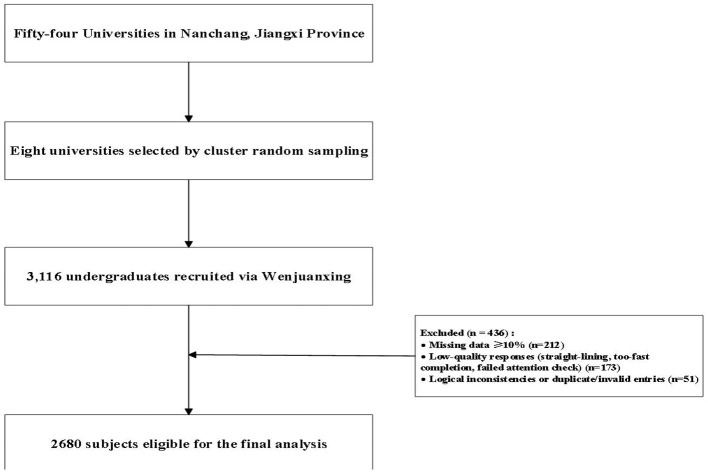
Recruitment and retention of participants.

An *a priori* power analysis using G^*^Power (*f*^2^ = 0.05, α = 0.05, 1–*β* = 0.90) indicated a minimum sample of 800. Considering the cluster design effect (DEFF = 1.2) and an anticipated 15% invalid-response rate, the target distribution was 1,100. To further improve the stability and precision of estimates in the moderated mediation model, and to ensure adequate representation across eight universities and multiple discipline categories, the research team intentionally recruited a larger sample than the minimum requirement (53). Larger samples are recommended for analyses involving interaction terms and multi-path effects and also help offset potential clustering influences beyond the estimated design effect. In total, 3,116 undergraduates were recruited via Wenjuanxing. As depicted in Figure 2, 436 cases were excluded based on pre-specified rules in three categories: (1) missing data ≥10% on core scales (*n* = 212); (2) low-quality responses (*n* = 173), defined as straight-lining within a scale, too-fast completion time (below the 1st percentile, calibrated via a pilot), or failing at least one attention-check item; and (3) logical inconsistencies or duplicate/invalid entries (*n* = 51), determined by contradictory demographics, repeated submissions identified via Wenjuanxing device/IP/account fingerprints, or non-authorized distribution channels. After exclusions, 2,680 participants were eligible for the final analysis (effective rate = 86%), meeting requirements for statistical power and representativeness. The valid sample included 1,277 males (47.6%) and 1,403 females (52.4%); mean age was 19.60 years (SD = 1.21). By grade: 878 freshmen (32.8%), 953 sophomores (35.6%), 677 juniors (25.3%), and 172 seniors (6.4%). By residence: 1,251 urban (46.7%) and 1,429 rural (53.3%).

Inclusion criteria were: full-time undergraduate enrollment; ability to read and understand Chinese; provision of electronic informed consent; and completion of the Wenjuanxing self-administered questionnaire. To enhance data quality, Wenjuanxing settings limited submissions to one per device/account/phone number, enabled IP anomaly alerts, randomized item order within scales, embedded 2–3 attention-check items and at least one reverse-scored item, and marked only core items as required. Exported datasets retained timestamps, completion time, source, and device information for quality control. All data were collected and stored anonymously under strict confidentiality, with access restricted to authorized team members. The study protocol was approved by the Ethics Committee of Jiangxi Normal University, and participation was entirely voluntary with the option to withdraw at any time.

### 2.2 Methods

#### 2.2.1 Independent variable: negative life events

This study measured negative life events using the Adolescent Self-Rating Life Events Checklist (ASLEC), a 26-item scale originally developed by Liu (54) and subsequently validated and updated by Xin (55). The ASLEC assesses the impact of life events across five dimensions: interpersonal stress, academic stress, punishment, loss, and adaptation. For each item, respondents first indicated whether the event occurred; if applicable, they then rated its impact on a six-point Likert scale, with higher scores reflecting greater negative impact. In the present sample, internal consistency was excellent (Cronbach's α = 0.942).

#### 2.2.2 Mediator: self-efficacy

Self-efficacy was measured using the general self-efficacy scale (GSES) developed by Schwarzer et al. (56). This self-report instrument assesses individuals' belief in their capability and persistence to accomplish required tasks. The questionnaire comprises 10 positively worded items, each rated on a four-point Likert scale from 1 (strongly disagree) to 4 (strongly agree). The total score theoretically ranges from 0 to 40, with higher scores indicating greater self-efficacy. The GSES has been widely used internationally, and empirical research indicates that the Chinese version demonstrates good reliability and validity in Chinese populations (16, 57). In the current study, internal consistency was good (Cronbach's α = 0.758).

#### 2.2.3 Moderator: physical activity

Physical activity was assessed using the physical activity rating scale (PARS), revised by Liang (58). Prior studies in Chinese samples have supported its reliability (59, 60). The scale evaluates three components—intensity, duration, and frequency—each rated on a scale of 5. The final score is computed as Intensity × (Duration – 1) × Frequency, with higher scores indicating higher overall physical activity. In the current study, internal consistency was good (Cronbach's α = 0.724).

#### 2.2.4 Dependent variable: problematic smartphone use

Problematic smartphone use was measured using the 10-item problematic smartphone use scale–short version (SAS-SV) (61). Each item is rated on a six-point Likert scale from 1 (strongly disagree) to 6 (strongly agree), yielding a total score ranging from 10 to 60, with higher scores indicating greater severity of problematic smartphone use. The scale covers five symptom domains: cyberspace-oriented relationship, overuse, and tolerance (one item each); daily-life disturbance (three items); and withdrawal (four items). For the multi-item domains, average scores were computed (range 1–6). The Chinese version of the problematic smartphone use Scale–Short Version has demonstrated good reliability and validity in prior research (62). In the present sample, internal consistency was good (Cronbach's α = 0.758). Although suggested cutoffs (male = 31; female = 33) can dichotomize scores into “non–problematic smartphone use” and “problematic smartphone use” (61), problematic smartphone use is not an official diagnosis but a continuum from non-problematic to problematic behavior (63); therefore, we used continuous problematic smartphone use scale–short version scores in all analyses.

### 2.3 Statistical analysis

Analyses were performed using IBM SPSS 26.0. First, continuous variables were compared between groups using independent-samples *t* tests or one-way ANOVA as appropriate; Dunnett's *t* test was applied for *post hoc* multiple comparisons. Second, Pearson correlation matrices were computed to describe associations among problematic smartphone use (SAS-SV), negative life events (ASLEC), self-efficacy (GSES), and physical activity (PARS); missing data were handled via multiple imputation. To examine the mechanism linking negative life events to SAS-SV, demographics showing significant univariate associations with SAS-SV were retained as covariates (categorical variables dummy-coded); the scores for ASLEC, GSES, and PARS were all mean-centered. Finally, the PROCESS macro (v3.5) with 5,000 bootstrap resamples was used to validate the models: in the simple mediation model (*X* = ASLEC, *M* = GSES, *Y* = SAS-SV), total, direct, and indirect effects with bias-corrected and accelerated 95% confidence intervals (BCa 95% CI) were reported; in the moderated mediation model, PARS served as the moderator to test the interaction of “negative life events × physical activity,” and conditional indirect effects were estimated at low/medium/high levels of PARS (mean ± 1 SD). Effects were deemed significant when the corresponding confidence intervals did not include zero. To harmonize scale metrics, total scores were standardized prior to modeling.

## 3 Results

### 3.1 Demographic characteristics

Participant demographics and between-group differences are presented in Table 1. Results from ANOVA and *t*-tests indicated that grade, sex, and residential area were significantly associated with negative life events, problematic smartphone use, and self-efficacy. In contrast, no significant differences were observed for physical activity. Accordingly, grade, sex, and residential area were treated as control variables in the subsequent analyses.

**Table 1:** Demographic characteristics of the study participants (*N* = 2,680) and univariate analyses of main study variables.

**Variables**	***n* (%)**	***t*/*F*-value**	***X***	***M***	***Y***	***Z***
**Grade**						
Freshman	878 (32.7)		47.87 ± 25.30	26.07 ± 5.84	33.74 ± 9.82	28.13 ± 30.30
Sophomore	953 (35.6)		42.38 ± 25.36	26.69 ± 6.35	35.51 ± 9.72	25.76 ± 29.89
Junior	677 (25.3)		48.10 ± 17.81	25.84 ± 4.74	34.04 ± 8.14	27.41 ± 29.50
Senior	172 (6.4)		44.56 ± 22.69	26.15 ± 5.70	34.05 ± 9.82	26.76 ± 30.42
		*F*-value	11.31^***^	3.24^*^	6.26^***^	1.10
**Sex**						
Male	1,277 (47.6)		44.58 ± 24.19	26.89 ± 5.63	32.48 ± 9.84	33.70 ± 31.46
Female	1,403 (52.4)		46.84 ± 23.05	25.64 ± 5.84	36.28 ± 8.62	20.94 ± 27.15
		*t*-value	−2.48^*^	5.61^***^	−10.60^***^	11.19
**Residential area**						
Urban	1,251 (46.7)		45.74 ± 22.14	26.22 ± 5.56	33.97 ± 9.62	27.67 ± 30.38
Rural	1,429 (53.3)		45.79 ± 24.86	26.26 ± 5.96	34.90 ± 9.21	26.44 ± 29.59
		*t*-value	−0.05	−0.18	−2.53^*^	1.06

X, negative life events; M, self-efficacy; Z, physical activity; Y, problematic smartphone use. ^*^p < 0.05; ^**^p < 0.01; ^***^p < 0.001.

### 3.2 Correlation analysis

Table 2 reports descriptive statistics and bivariate correlations. Negative life events were positively associated with problematic smartphone use and negatively associated with both self-efficacy and physical activity. Self-efficacy showed a significant negative correlation with problematic smartphone use and a positive correlation with physical activity. Physical activity was negatively correlated with problematic smartphone use.

**Table 2:** Descriptive statistics and bivariate correlations among the main study variables. *N* = 2,680.

**Variables**	**Mean**	**SD**	**1**	**2**	**3**	**4**
1. *X*	45.76	23.62	1			
2.*M*	26.24	5.78	−0.25^**^	1		
3. *Y*	34.47	9.41	0.27^**^	−0.29^**^	1	
4. *Z*	27.02	29.96	−0.12^**^	0.23^**^	−0.58^**^	1

X, negative life events; M, self-efficacy; Z, physical activity; Y, problematic smartphone use. ^*^p < 0.05; ^**^p < 0.01; ^***^p < 0.001.

### 3.3 Mediation analysis

**Figure 3:**
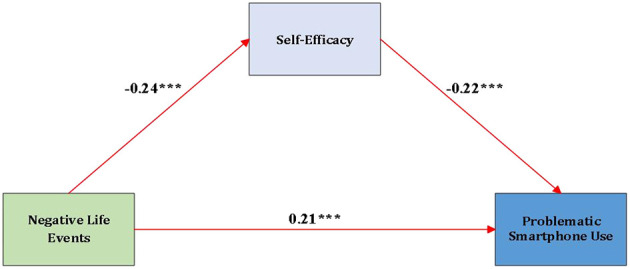
Mediation effect diagram. ***p < 0.001.

We assessed multicollinearity via the Variance Inflation Factor; the maximum value was 1.11, indicating no concern. Variables were standardized. Using Hayes's PROCESS macro for SPSS (Model 4) and controlling for grade level, residential background, and sex, negative life events were associated with lower self-efficacy (*β* = −0.24, *t* = −12.97, *p* < 0.001) and higher problematic smartphone use (*β* = 0.21, *t* = 11.40, *p* < 0.001) (Table 3). Self-efficacy was inversely associated with problematic smartphone use (*β* = −0.22, *t* = −12.23, *p* < 0.001). The indirect association of negative life events with problematic smartphone use via self-efficacy was 0.05 [95% bootstrap CI (0.04, 0.07)]. Self-efficacy partially mediates the association (see Figure 3). These demographic variables were included as covariates because prior research indicates that developmental differences across grade levels, gender-related variations in emotional and behavioral regulation, and disparities in stress exposure and resource availability between urban and rural contexts may all influence smartphone use, self-efficacy, and the experience of negative life events (64–67). Controlling for these factors helps isolate the unique effects of the primary study variables.

**Table 3:** Regression results for problematic smartphone use. *N* = 2,680.

**Variables**	***M***		***Y***	
	*β*	***t***	*β*	***t***
Sex	−0.19	−5.14^***^	0.14	4.56^***^
Grade	−0.01	−0.58	0.01	0.21
Resident	0.01	0.23	0.08	2.71^*^
*X*	−0.24	−12.97^***^	0.26	12.81^***^
*M*			−0.12	−7.93^***^
*X* ^*^ *Z*			−0.01	−6.29^***^
*R* ^2^	0.07		0.41	
*F*	50.49^***^		268.24^***^	

X, negative life events; M, self-efficacy; Z, physical activity; Y, problematic smartphone use. ^*^p < 0.05; ^**^p < 0.01; ^***^p < 0.001.

### 3.4 Moderation analysis

Using Hayes's PROCESS macro for SPSS (Model 5) and controlling for grade level, residential background, and sex, negative life events were positively associated with problematic smartphone use (*β* = 0.26, *t* = 12.81, *p* < 0.001). In contrast, self-efficacy was negatively associated with problematic smartphone use (*β* = −0.12, *t* = −7.93, *p* < 0.001). The interaction between negative life events and physical activity was negatively associated with problematic smartphone use (*β* = −0.01, *t* = −6.29, *p* < 0.001), indicating a moderating effect whereby the positive association between negative life events and problematic smartphone use is weaker at higher levels of physical activity.

**Figure 4:**
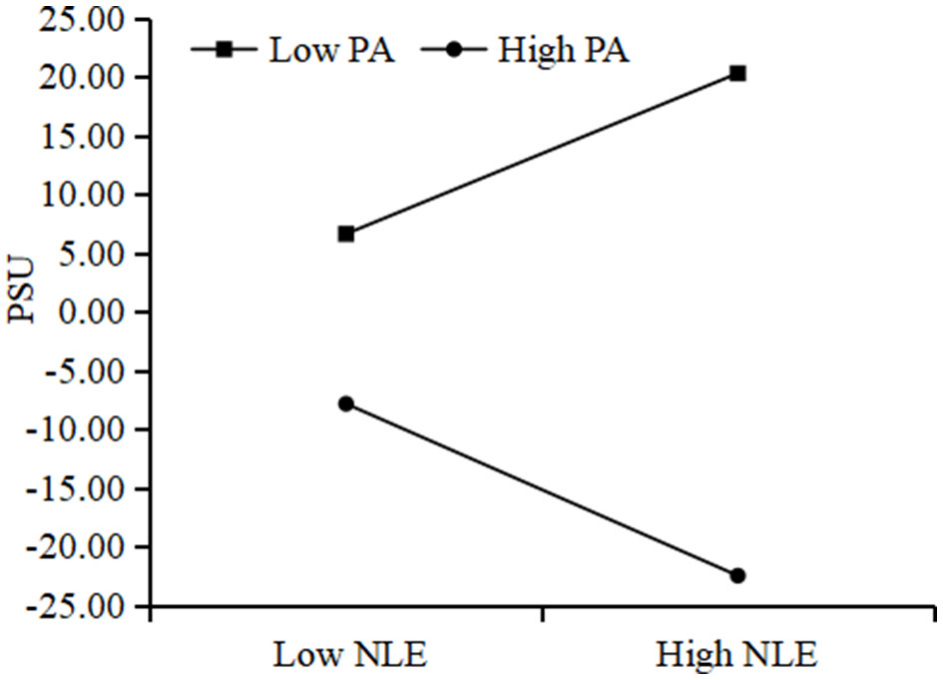
Moderation effect test.

To illustrate the moderated mediation model, we conducted a simple-slopes analysis, defining high and low physical activity groups as one standard deviation above and below the mean, respectively. This analysis tested whether physical activity moderates the association between negative life events and problematic smartphone use. As shown in Figure 4, the association was stronger in the low physical activity group (*β* = 0.26, *t* = 12.81, *p* < 0.001) and weaker in the high physical activity group (*β* = 0.09, *t* = 4.36, *p* < 0.001).

## 4 Discussion

Problematic smartphone use, a maladaptive behavior, has drawn sustained attention in mental health research (68). Prior work with school-aged samples links it to poorer social–emotional outcomes (69). Guided by social learning theory, this study probed how online negative life events relate to problematic smartphone use in university students, focusing on mechanisms and protective factors (70). We found that self-efficacy partially mediated the association between negative life events and problematic smartphone use, and that physical activity weakened the pathway by moderation (16). These results inform theory and point to intervention targets for university populations in the digital era (71).

### 4.1 Negative life events and problematic smartphone use among university students

First, there is a significant positive association between negative life events and indicators of problematic smartphone use among university students, a finding that aligns with prior research (72). In university life, negative events such as academic setbacks, interpersonal conflicts, and family difficulties are relatively common (73). Related studies indicate that these events frequently co-occur with experiences of stress, anxiety, and helplessness (74). Because smartphones are convenient and can provide immediate attentional distraction, they are often used for emotion regulation or short-term comfort (75). Evidence shows that higher frequencies of entertainment, gaming, and social media use covary with stronger indicators of problematic smartphone use (76). When individuals simultaneously experience negative affect and perceived discrepancies between goals and reality, they may be more inclined to engage in immersive phone use to obtain short-term pleasure, which is associated with heightened indicators of problematic smartphone use (77).

According to stress and coping theory, individuals appraise stressors and select coping strategies when facing negative life events (78). Students with lower self-efficacy and more avoidance-oriented coping styles are often observed to display greater attentional shifting and online escapism, which are associated with more intensive phone use and indicators of problematic smartphone use (79). Within group contexts and under peer norms, some students, even without an explicit intention to increase online engagement, may exhibit more frequent phone use in a go-with-the-flow pattern, reflecting the indirect influence of social contexts on individual digital behavior (80).

In the current learning and evaluation ecology, greater emphasis on digitalized learning requirements, online assignment submissions, and the use of social collaboration platforms has blurred the boundaries between academics and social life (81). A performance-oriented and visibility-oriented learning and social environment may be associated with more frequent online participation, a stronger pursuit of immediate feedback, and experiences of fear of missing out (82).

### 4.2 The mediating role of self-efficacy

Second, the findings indicate a partial mediation by self-efficacy in the link between negative life events and problematic smartphone use. Specifically, greater exposure to negative life events is associated with lower self-efficacy, which, in turn, is related to higher problematic smartphone use (83, 84). Social learning theory states that self-efficacy, defined as one's belief in the ability to organize and carry out actions, influences behavioral choices, effort, and persistence (85, 86). When university students encounter negative life events, those with higher self-efficacy are more likely to cope actively, search for effective solutions, and avoid relying on smartphones as a means of escape (87).

Self-efficacy can be regarded as a psychological resource that facilitates positive attitudes and adaptive coping under stress (88). Students with high self-efficacy are more confident in overcoming difficulties and thus tend to adopt proactive coping strategies such as seeking social support, adjusting goals, or reorganizing time, reducing their reliance on smartphones (89). Conversely, students with low self-efficacy may feel helpless and confused when facing life stressors, lacking confidence to cope, and are therefore more likely to resort to problematic smartphone use as a maladaptive coping strategy (90, 91).

This finding echoes previous research. Negative life events can produce adverse effects on psychological health, such as anxiety and depression, which further weaken self-efficacy (92). Once self-efficacy is impaired, individuals are trapped in a cycle of negative emotions, increasing the risk of problematic smartphone use (93). For example, a study found that university students' self-efficacy significantly declined following interpersonal conflicts, and this reduction was positively associated with higher problematic smartphone use (94). Another study also suggested that self-efficacy mediated the link between negative life events and problematic smartphone use, and strengthening students' self-efficacy could effectively alleviate addiction risk (95, 96).

### 4.3 The moderating role of physical activity

Third, physical activity moderated the link between self-efficacy and problematic smartphone use, with the effect most evident among students with lower self-efficacy. At low levels of self-efficacy, higher physical activity weakened the association, such that self-efficacy no longer significantly predicted problematic smartphone use (97, 98). According to relevant theories, physical activity, including aerobic exercise and sports participation, enhances physical fitness and positively affects psychological wellbeing (99).

When individuals encounter negative life events and suffer setbacks in self-efficacy, engaging in physical activity can help relieve stress and improve emotional states, thereby reducing or preventing problematic smartphone use (100). Exercise stimulates neurotransmitters such as dopamine and endorphins that elevate mood and lessen anxiety and depressive symptoms (101, 102). Accordingly, among more physically active students, the association between lower self-efficacy and higher problematic smartphone use is no longer significant (103).

Physical activity can also directly enhance self-efficacy, as confirmed by the positive correlation between the two in this study. Students with higher levels of physical activity may possess better self-regulation, enabling them to offset the adverse consequences of diminished self-efficacy (104). Moreover, physical activity improves both physical condition and psychological resilience, strengthening adaptive coping under stress (50). Among various behavioral responses to negative life events, physical activity is regarded as the most socially adaptive, as it allows individuals to benefit from stress without undermining their mental health (105). Thus, students who engage in more physical activity may rely on it as a strategy to buffer the effect of reduced self-efficacy on problematic smartphone use.

### 4.4 Limitations

Several limitations should be acknowledged. To begin with, the sample comprised only university students. Problematic smartphone use is observed across diverse age groups, and developmental stages may shape how negative life events, self-efficacy, and physical activity relate to use patterns. Broadening the sampling frame to include adolescents, middle-aged adults, and older adults would enable tests of age-related heterogeneity. In addition, the cross-sectional design restricts inferences about temporal ordering. Although associations were identified, the direction and dynamics of change remain unclear. Adopting longitudinal designs with multiple waves, experience sampling, or micro-longitudinal approaches could clarify within-person processes and time-lagged relations. Where ethical and feasible, quasi-experimental designs and intervention trials may further strengthen causal inference without relying solely on observational data.

Moreover, the study emphasized the mediating role of self-efficacy and the moderating role of physical activity, while other variables were not modeled. Family environment, social support, personality traits, sleep quality, academic demands, coping styles, and digital platform features may also be relevant to problematic smartphone use. Incorporating a broader set of individual, interpersonal, and contextual factors and testing alternative or competing models would yield a more integrated account. A further constraint concerns sampling scope. Participants were recruited from eight universities in Nanchang, Jiangxi Province, which may limit generalizability to other regions and cultural settings. Multi-site sampling across provinces, institution types, and urban–rural contexts, as well as cross-cultural comparisons, could enhance external validity.

Methodologically, reliance on self-report measures introduces risks of common method variance, recall bias, and social desirability effects. Combining self-reports with objective behavioral logs, passive sensing, peer or teacher reports, and performance-based tasks would mitigate shared-method bias and improve measurement precision. Measurement issues also warrant attention. Some constructs were assessed using brief scales, which may constrain reliability and content coverage. Future work could employ validated multidimensional instruments, conduct measurement invariance testing across subgroups, and apply modern psychometric methods, such as item response theory, to refine indices of problematic smartphone use.

Finally, unobserved confounding remains possible. Variables such as pre-existing mental health conditions, socioeconomic status, campus policy environments, or regional digital infrastructure might correlate with both negative life events and problematic smartphone use. Incorporating these covariates, using propensity-based approaches, or applying within-person fixed-effects models where data permit, may reduce bias in estimated associations.

### 4.5 Implications

The present findings extend our understanding of how negative life events relate to problematic smartphone use during youth and highlight potential avenues for prevention and intervention within university settings. Based on these results, several practical recommendations can be proposed to reduce problematic smartphone use among university students.

First, it is crucial to identify students who have recently experienced negative life events and who show atypical patterns of phone use. Universities should establish screening and intervention mechanisms that combine annual mental health surveys, brief interviews, and standardized questionnaires to identify individuals at higher risk. Based on screening results, tiered psychological support can be implemented. For higher-risk students, provide cognitive behavioral counseling to help restore a sense of control and strengthen self-regulation strategies. For lower-risk students who show signs of overuse, organize group-based development programs such as digital hygiene workshops. In addition, collaborate with external agencies to develop intervention programs and build an integrated service network that covers screening, intervention, follow-up, and evaluation to support students' digital health.

Second, strengthening self-efficacy is essential. Campus mental health professionals should develop psychoeducation and skills training to help students build confidence in academic, social, and self-regulatory domains when facing challenges. Workshops focused on mastery-oriented goal-setting, problem-solving, and cognitive reappraisal can help reduce maladaptive phone use. At the same time, reinforce peer support networks by organizing peer mentoring programs, study groups, and mental health activities to cultivate interpersonal connections and a sense of responsibility, foster a supportive campus culture, buffer stress, and improve self-regulation.

Finally, promoting regular physical activity is an important strategy for enhancing psychological resilience and lowering the risk of problematic smartphone use. Regular physical activity improves physical health and provides psychological benefits by facilitating the release of dopamine and endorphins, improving sleep quality, and stabilizing mood. Universities should integrate physical activity into mental health initiatives and create comprehensive intervention models. Counseling centers can collaborate with physical education departments to provide mindfulness-informed exercise programs, such as attention-focused yoga and mindful running groups, to strengthen self-control. Implement activity-tracking platforms and incentive mechanisms, encourage students to form sports clubs or outdoor activity groups, and cultivate positive social connections that provide offline alternatives to passive screen use. By building a campus culture that values physical activity, universities can enhance students' self-efficacy and psychological resilience and promote healthier engagement with technology.

## 5 Conclusions

This study examined the association between negative life events and problematic smartphone use among university students, testing the mediating role of self-efficacy and the moderating role of physical activity. Findings indicated a positive association between negative life events and problematic smartphone use, with self-efficacy partially mediating this link and physical activity moderating the path from self-efficacy to problematic smartphone use. The study underscores multilayered influences and suggests practical directions involving screening, psychoeducation, and integrating physical activity on campus. These conclusions are based on correlational evidence, highlighting the need for longitudinal and multi-source data to strengthen internal and external validity.

## Data Availability

The original contributions presented in the study are included in the article/supplementary material, further inquiries can be directed to the corresponding authors.
